# Stage-Specific Germ-Cell Marker Genes Are Expressed in All Mouse Pluripotent Cell Types and Emerge Early during Induced Pluripotency

**DOI:** 10.1371/journal.pone.0022413

**Published:** 2011-07-25

**Authors:** Xingbo Xu, D. V. Krishna Pantakani, Sandra Lührig, Xiaoying Tan, Tatjana Khromov, Jessica Nolte, Ralf Dressel, Ulrich Zechner, Wolfgang Engel

**Affiliations:** 1 Institute of Human Genetics, University of Goettingen, Goettingen, Germany; 2 Department of Cellular and Molecular Immunology, University of Goettingen, Goettingen, Germany; 3 Institute of Human Genetics, University of Mainz, Mainz, Germany; Instituto Nacional de Câncer, Brazil

## Abstract

Embryonic stem cells (ESCs) generated from the *in-vitro* culture of blastocyst stage embryos are known as equivalent to blastocyst inner cell mass (ICM) *in-vivo*. Though several reports have shown the expression of germ cell/pre-meiotic (GC/PrM) markers in ESCs, their functional relevance for the pluripotency and germ line commitment are largely unknown. In the present study, we used mouse as a model system and systematically analyzed the RNA and protein expression of GC/PrM markers in ESCs and found them to be comparable to the expression of cultured pluripotent cells originated from the germ line. Further, siRNA knockdown experiments have demonstrated the parallel maintenance and independence of pluripotent and GC/PrM networks in ESCs. Through chromatin immunoprecipitation experiments, we observed that pluripotent cells exhibit active chromatin states at GC marker genes and a bivalent chromatin structure at PrM marker genes. Moreover, gene expression analysis during the time course of iPS cells generation revealed that the expression of GC markers precedes pluripotency markers. Collectively, through our observations we hypothesize that the chromatin state and the expression of GC/PrM markers might indicate molecular parallels between *in-vivo* germ cell specification and pluripotent stem cell generation.

## Introduction

The capacity of long-term self-renewal and the unique ability to differentiate into all three germ layer cell types (ectoderm, endoderm and mesoderm) define pluripotency. According to the source of cells used for the establishment of pluripotent cells, one can currently distinguish between the following pluripotent cell lines: (1) embryonic stem cells (ESCs) derived from the inner cell mass of mouse blastocysts at embryonic day 3.5 (E3.5) [1], [2], (2) embryonic germ cells (EGCs) cultured from primordial germ cells (PGCs) that colonize the genital ridge at E12.5 [3], [4], (3) embryonal carcinoma cells (ECCs) isolated from germ-cell tumors of either testis or ovary [5], (4) germ line stem cells isolated from mouse neonatal and adult testis (GSCs and maGSCs, respectively) [6], [7] and (5) induced pluripotent stem cells (iPSCs), derived from reprogramming somatic cells by ectopic expression of defined transcription factors [8]. All the above mentioned cultured pluripotent cell lines (EGCs, ECCs, GSCs, and maGSCs) have a germ-cell origin, except ESCs, whose origin is not clearly understood. Although these cell lines have different molecular profiles mostly due to their developmental stage of isolation, they share the expression of germ cell/pre-meiotic (GC/PrM) markers that may indicate a germ-cell origin [9].

During embryonic development, the specification of PGCs is crucial for the development of the germ line, which is finally destined to give rise to the totipotent zygote upon fertilization. Prior to gastrulation, the precursors of primordial germ cells arise in the E6.25 proximal epiblast from 4–8 cells positive for the transcriptional repressor Blimp1 [10], [11]. These Blimp1-positive cells continuously proliferate and start to express other PGC markers such as Fragilis and Stella by E7.5. Thereafter, PGCs initiate migration and colonization of the genital ridge and increase their number to approximately 4000 by E12.5 [12], [13]. Further development of PGC/germ cells to mature spermatozoa or oocytes depends on the coordinated genetic and epigenetic events [14]. Interestingly, several studies have demonstrated the expression of some of the GC/PrM markers like Blimp1, Stella, Fragilis, Piwil2, Dazl and MVH in ES cells at the RNA level [15], [16], [17], raising the possibility that ES cells might originate from the germ line [9].

In the present study, using mouse as a model system, we have systematically analyzed the expression of GC/PrM markers in ES cells compared to germ line origin cultured pluripotent cells like EGCs, ECCs, GSCs and maGSCs and found comparable expression at the RNA and protein level. Moreover, we show the expression of Stella, Dazl and MVH in preimplantation embryos and, the independence of pluripotency-specific networks from germ cell-specific networks in ES cells. Interestingly, chromatin immunoprecipitation (ChIP) analysis revealed that ES cells exhibit active chromatin states at GC marker genes and a bivalent chromatin structure at PrM marker genes. Further, gene expression analysis during iPSC generation revealed that the expression of GC markers precedes pluripotency markers. Collectively, our data indicates the possible link between *in-vivo* germ cells specification and *in-vitro* pluripotent stem cells generation.

## Materials and Methods

### Cell culture

Derivation and maintenance of male mouse ESC and maGSC lines from different genetic backgrounds (129Sv and C57BL/6) were described previously [18]. The female ESC line ES Rosa26 was generated from Rosa26-LacZ transgenic mouse line as described for MPI-VI, a previously generated female ESC line [19]. iPS cells (O18) were a kind gift from Prof. Rudolf Jaenisch [20]. All above cell lines including EGC line (EG 1) and parthenogenetic cells were maintained in standard ESC culture conditions. ECC cell line (F9) protein extract was provided by Mr. Peter Christalla, Goettingen. For knockdown experiments, ES cells were seeded in KO-DMEM supplemented with KO-serum replacement (Invitrogen) at a density of 2×10^5^ cells/ml on feeder layer. After 5 h of plating, the cells were transfected with either *Dazl* siRNA (NM_010021.4_stealth_199, _726, _1056, Invitrogen) or *MVH* siRNA (NM_001145885.1_stealth_83, _922, _1599, Invitrogen) or *Oct3/4* siRNA (NM_013633_Stealth_356, _463, _727, Invitrogen) or scrambled siRNA (Control siRNA, Invitrogen) using Lipofectamine-2000 (Invitrogen) according to manufacturer's instructions. After 3 h of transfection, the medium was changed to standard ES culture medium and allowed to grow for 24 h. The next day, transfection was repeated and cells were harvested after additional 24 h of culture.

All animal experimentations were reviewed and approved by the Institutional Animal Care and Use committee of the University of Goettingen (Approval ID: 33.9.42502-097/06).

### Generation of iPS cells

We used Yamanaka factors (retroviral expression vectors for Oct3/4, Sox2, Klf4, c-Myc) to generate iPS cells [8]. For reprogramming studies, embryonic fibroblasts isolated from transgenic *Nanog*-EGFP mice [21] were transduced with retroviral particles as previously described [8]. To establish iPS cell lines, colonies which appeared after 10 days of virus infection were picked manually and cultured in 24-well plates containing feeder layer with standard ES medium and were monitored for the *Nanog*-EGFP activation using invert microscope (Olympus). For time course experiments, all cells from culture plates were collected on days 0 (Control), 5-10, 12, 14, 16, 18, 20 and 22 after viral infection for total RNA isolation.

### RNA extraction, RT-PCR and qPCR

Total RNA was extracted from cells using Trizol Reagent (Invitrogen) and from ∼50 blastocysts using Picopure Kit (Analytical Technology) following the manufacturers' protocols. Total RNA from blastocysts or 5 µg of total RNA from cells was digested with *DNaseI* (Sigma) and used for cDNA synthesis using the SuperScriptII system (Invitrogen). For qPCR analysis, diluted cDNA (1/20) was used as a template in a Platinum SYBR Green qPCR SuperMix-UDG with ROX (Invitrogen) and run in ABI 7900HT Real-Time PCR System (Applied Biosystems). Primers used in RT-PCR and qPCR are listed in supplementary tables (Tables S1, S2, S3).

### Protein extraction and Western blotting

Proteins were extracted from cells and tissues using lysis buffer (10 mM Tris–HCl (pH 8.0), 1 mM EDTA, 2.5% SDS, 100 mM PMSF) containing protease inhibitor cocktail (Roche). Protein samples were resolved on 4–12% SDS-PAGE and transferred onto nitrocellulose membrane (Amersham Biosciences). Membranes were processed using standard Western blot protocols, and signals detected using a chemiluminescent kit (Santa Cruz Biotechnology). Antibody sources are listed in supplementary tables (Table S5).

### Early-stage-embryo collection, immunocytochemistry, and alkaline phosphatase staining

Early stage embryos collected from the oviduct or uterus of pregnant CD-1 mice were used for whole-mount immunostaining as described previously [22]. Immunostained embryos were mounted on cavity microscope slides using Vectashield DAPI mounting medium (Vector Laboratories) and images were acquired using an Olympus confocal microscope. Immunostaining on iPS cells using SSEA1 antibody was performed as previously described [23]. Alkaline phosphatase **(**AP) staining was performed according to manufacturer's protocol (Sigma).

### Chromatin immunoprecipitation

The chromatin immunoprecipitation (ChIP) was performed essentially as previously described [23]. Briefly, cultured wild-type ESCs were cross-linked with 1% formaldehyde and briefly incubated in lysis buffer followed by sonication (Branson Sonifier 250). Soluble chromatin was pre-cleared with protein-A sepharose beads and incubated with and without (negative-control) antisera (3–5 µg) directed against H3K4me3, H3K9ac, H3K9me3, and H3K27me3. Enriched DNA was analyzed by qPCR using SYBR green (Invitrogen)-based PCR amplification with primers listed in supplementary tables (Table S4). qPCR data from two or more biological replicates were calculated and expressed as percentage of input DNA precipitation. Antibodies used in ChIP are listed in supplementary tables (Table S5).

### Teratoma formation assay

Teratoma formation assay was performed as previously described [24], [25]. Briefly, iPS cells (1×10^6^ cells) were injected subcutaneously into 8 to 10 weeks old female severe combined immunodeficient RAG2^−/−^cγc^−/−^ mice lacking T, B, and natural killer (NK) cells. Tumor growth was monitored weekly by palpation and size was recorded using linear calipers. Animals were sacrificed when a tumor diameter of 1 cm was reached. Autopsies were performed and tumor tissue was placed in phosphate-buffered 4% formalin for 16 h and then embedded in paraffin. For histological analysis, the specimens were stained with haematoxylin and eosin (HE).

### Statistical Analysis

All qPCR data for RNA expression analysis (two or more biological replicates) were calculated using standard curve method. For statistical significance calculations, 2way ANOVA (GraphPad Prism 4.0) test was used.

## Results

### Pluripotent stem cells express GC/PrM genes

**Figure 1:**
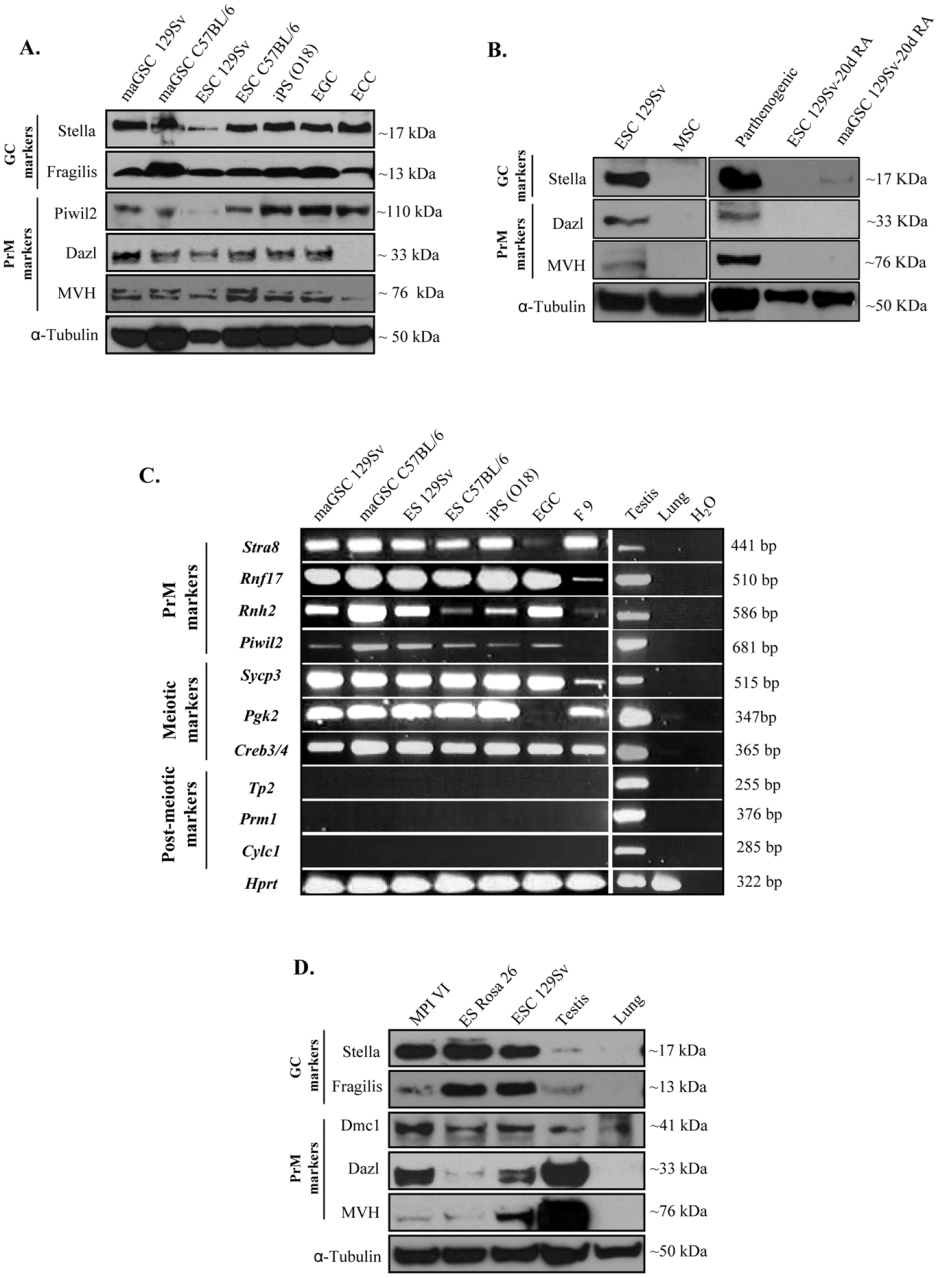
Expression analysis of GC/PrM genes in pluripotent cell lines. (**A**) Expression of GC markers (Stella, Fragilis) and PrM markers (Piwil2, Dazl, MVH) in maGSCs and ESCs (of 129Sv and C57BL/6 genetic backgrounds), iPSCs, EGCs and ECCs**.** (**B**) Western blot of GC marker (Stella) and PrM markers (Dazl and MVH) in mouse mesenchymal stromal cells (MSC), parthenogenetic cells, ES and maGSC treated with retinoic acid for 20 days. Protein extract from ESCs was used as a positive control. (**C**) RT-PCR analysis showing the expression of PrM (*Stra8*, *Rnf17*, *Rnh2* and *Piwil2*), meiotic (*Sycp3*, *Pgk2* and *Creb3/4*) and post-meiotic (*Tp2*, *Prm1* and *Cylc1*) markers in maGSC and ESC (from different genetic backgrounds), as well as iPSC, EGC and ECC cell lines. cDNA from wild type mouse testis and lung were used as positive and negative controls, respectively. (**D**) Western blot analysis showing the expression of GC markers (Stella, Fragilis), PrM markers (Dmc1, Dazl, MVH) in two different female ESC lines (MPI VI, ES Rosa26). α-tubulin was used as loading control in **A, B and D**.

To investigate whether GC/PrM gene expression is characteristic of all known mouse pluripotent cells, we systematically analyzed several pluripotent cells. Firstly, we examined GC/PrM gene expression in male maGSCs and ESCs from different genetic backgrounds and in iPSCs, EGCs, and F9 cells by Western blotting (Fig. 1A). GC markers Stella and Fragilis were readily detected in all cell types (Fig. 1A), including parthenogenetic cells (Fig. 1B). Further, PrM markers Piwil2, Dazl, and MVH were found to be expressed in all pluripotent cells, except ECCs (Fig. 1A). Protein levels of GC/PrM markers were reduced or absent in ESCs and maGSCs upon spontaneous differentiation with retinoic acid for 20 days (Fig. 1B). Overmore, we analyzed multipotent mesenchymal stem cells (MSCs) and could not detect any expression of GC/PrM markers (Fig. 1B). We also performed RT-PCR analysis for other PrM, meiotic, and post-meiotic markers (Fig. 1C). Expression of PrM markers *Stra8*, *Rnf17*, *Rnh2*, and *Piwil2* was detected in all cells (Fig. 1C). Surprisingly, meiotic markers *Sycp3*, *Pgk2*, and *Creb3/4* were also detected in all pluripotent cell lines (Fig. 1C). However, expression of several other developmental markers such as post-meiotically expressed *Tp2*, *Theg*, *Gpx4*, *Prm1*, and mature spermatozoan marker *Cylc1* was undetectable as expected (Fig. 1C).

To determine whether the expression of GC/PrM markers is specific to male pluripotent cells, we studied two female ES cell lines, namely, MPI VI and ES Rosa26. Pluripotency of these cell lines was confirmed by detecting the expression of the key pluripotency markers Oct3/4 and Sox2 (data not shown). Both female pluripotent cell lines were found to express all analyzed GC/PrM markers with levels similar to those of male pluripotent cells (Fig. 1D).

### GC/PrM genes are also expressed in early embryogenesis

**Figure 2:**
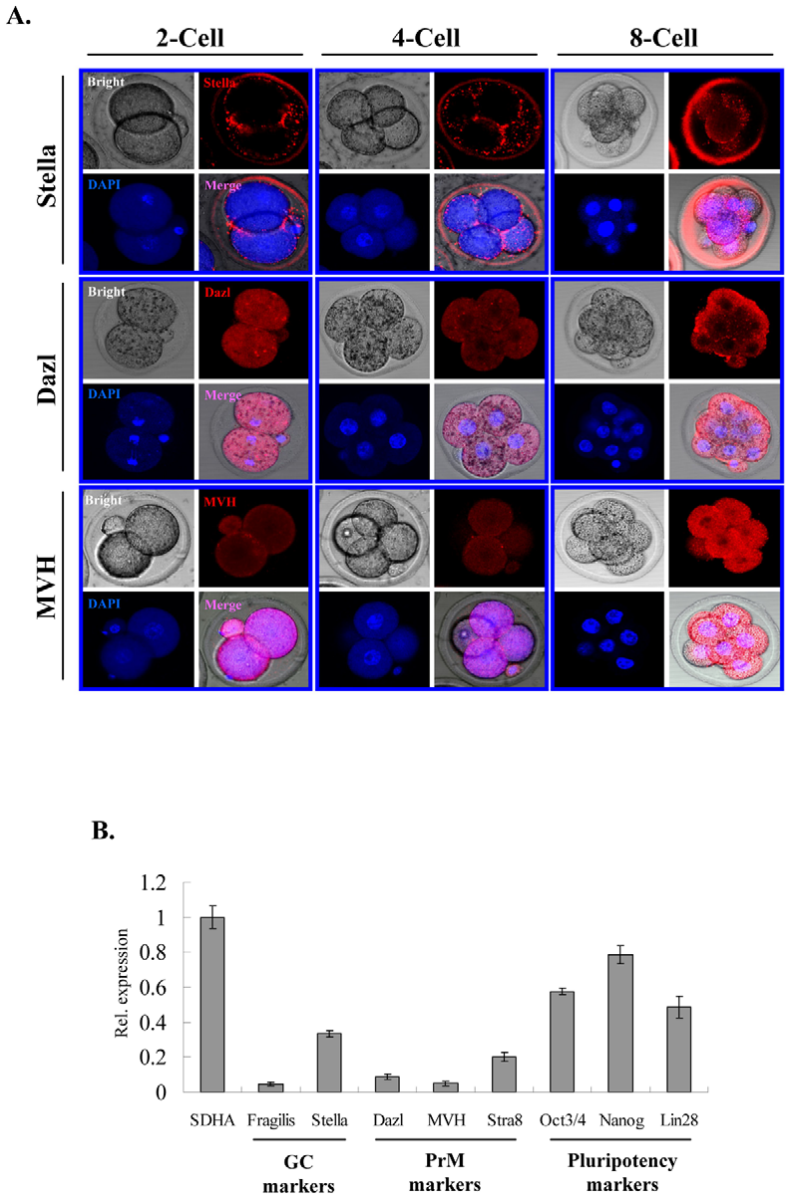
Expression analysis of GC/PrM genes in early embryogenesis. (**A**) Immunocytochemistry showing the expression of GC marker (Stella) and PrM markers (Dazl and MVH) in pre-implantation embryos (2-cell, 4-cell, and 8-cell stage). DAPI was used as a counter stain to visualize the nucleus. (**B**) Quantitative real time PCR analysis was used to evaluate the expression levels of GC (Fragilis), PrM (Dazl, MVH, Stra8) and pluripotency markes (Oct3/4, Nanog, Lin28) in blastocysts. Expression levels were normalized to *Sdha* (a house keeping gene). The qPCR data of three biological replicates (including three technical replicates each) were calculated and represented as a mean ±SD.

Next, we studied the expression of GC marker (Stella) and PrM markers (Dazl and MVH) in early stages of mouse embryogenesis (2-, 4-, 8-cell stages) by immunocytochemistry (ICC) (Fig. 2A). Interestingly, we found Stella, Dazl and MVH to be expressed throughout all stages of embryogenesis (Fig. 2A). To determine the expression levels of GC/PrM markers at the blastocyst stage, we performed qPCR on blastocyst stage embryos (Fig. 2B). In agreement with our ICC results, all analyzed GC/PrM markers (*Fragilis*, *Dazl*, *MVH*, and *Stra8*) were detected at the blastocyst stage with transcript levels, that are, however, markedly lower than those of pluripotency markers such as *Oct3/4*, *Nanog*, *Lin28* (Fig. 2B).

### Independency of pluripotent and GC/PrM networks in ESCs

**Figure 3:**
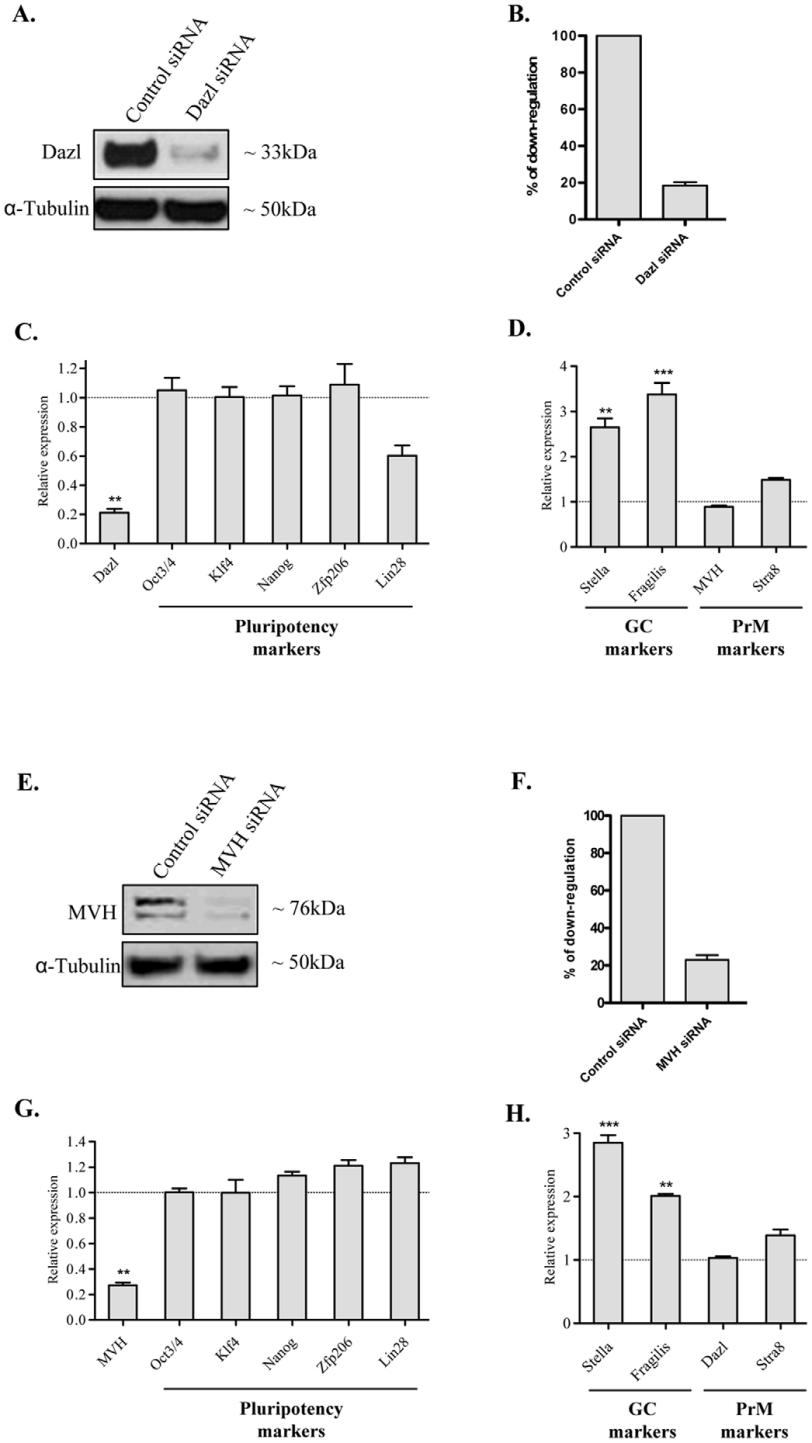
Effect of the downregulation of PrM genes in ESCs. (**A and B**) Efficiency of Dazl down-regulation as shown by Western blot analysis and subsequent densitometric qunatification**.** (**C**) Real time qPCR analysis showing *Dazl* downregulation at the RNA level and the expression profile of the core pluripotency network (*Oct3/4*, *Klf4*, *Nanog*, *Zfp206* and *Lin28*). (**D**) Expression of GC (*Stella* and *Fragilis*) and PrM (*MVH* and *Stra8*) markers in *Dazl* down-regulated ESCs. (**E and F**) Western blot showing the efficiency of MVH downregulation and the densitometric quantification, respectively. (**G**) Real time qPCR analysis showing *MVH* downregulation at the RNA level and the expression profile of the core pluripotency network (*Oct3/4*, *Klf4*, *Nanog*, *Zfp206* and *Lin28*). (**H**) Expression of GC (*Stella* and *Fragilis*) and PrM (*Dazl* and *Stra8*) markers in *MVH* down-regulated ESCs. The dotted lines in **C**, **D**, **G**, **H** indicate the normalized expression levels of analyzed genes in control siRNA treated cells. The qPCR data of two biological replicates (including three technical replicates each) were calculated and represented as a mean ±SD. Expression levels, which are statistically significant, are indicated with asterisks (**p<0.01; ***p<0.001).

The widespread expression of GC/PrM markers in pluripotent cells led us to study their influence on other GC/PrM and key pluripotency markers (Fig. 3). Firstly, we down-regulated *Dazl* in ES cells using siRNA and found an ∼80–90% decrease at both the RNA and protein level (Fig. 3A, B). In contrast, control siRNA treated cells did not exhibit altered Dazl expression levels (Fig. 3A, B). Then, we performed a qPCR-based analysis of expression levels of key pluripotency markers and detected no significant differences among control siRNA treated and Dazl siRNA treated cells (Fig. 3C). Similarly, the expression of PrM markers *MVH* and *Stra8* did not change significantly, whereas GC markers (*Stella* and *Fragilis*) showed significant up-regulation in Dazl down-regulated cells (Fig. 3D). Similarly, ∼70% down-regulation of *MVH* expression (Fig. 3E, F) did not influence expression of key pluripotency markers (Fig. 3G). Consistent with Dazl down-regulation, MVH depletion had no effect on *Dazl* and *Stra8*, but *Stella* and *Fragilis* were significantly up-regulated (Fig. 3H). Conversely, we down-regulated *Oct3/4* and studied the expression of GC/PrM and pluripotency markers. The down-regulation of *Oct3/4* resulted in significant down-regulation of *Klf4* expression, whereas the expression of other pluripotent markers such as *Nanog*, *Zfp206*, and *Lin28* did not alter (Supplementary Fig. S1). Furthermore, the down-regulation of *Oct3/4* had no statistically significant effect on the expression of GC/PrM markers (Supplementary Fig. S1).

### Active chromatin at GC marker gene promoter regions and bivalent chromatin at PrM marker gene promoters in ESCs and iPSCs

**Figure 4:**
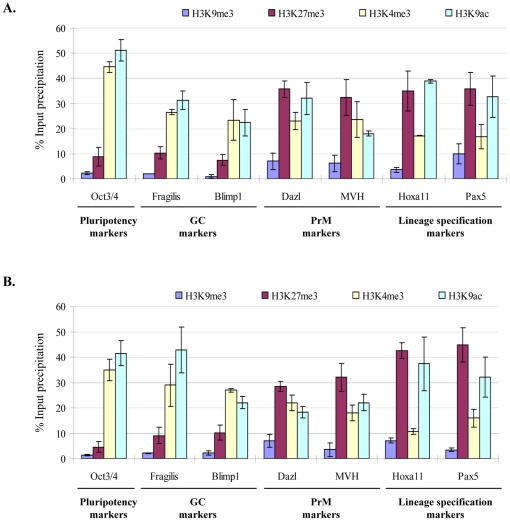
Epigenetic signature of GC/PrM genes in ESCs and iPSCs. ChIP and subsequent real time qPCR for various histone modifications (active: H3K4me3 and H3K9ac; repressive: H3K9me3 and H3K27me3) at the promoter regions of GC (*Fragilis* and *Blimp1*) and PrM markers (*Dazl* and *MVH*) in ESCs (**A**) and iPSCs (**B**). The promoter regions of GC markers are similar to the *Oct3/4* promoter and are enriched for active histone modifications, while the promoters of PrM markers are similar to the promoters of the lineage specific genes *Hoxa11*, *Pax5* and were enriched for both active and repressive marks indicating their bivalent chromatin structure. The qPCR data of two biological replicates (including three technical replicates each) were calculated and represented as a mean ±SD.

We hypothesized that the chromatin state at the promoter regions of GC/PrM markers might elucidate their role in the establishment/maintenance of pluripotency or lineage specification in ESCs. We analyzed the ChIP sequencing data of mouse ES cells, which is freely available [26] and found that the promoter regions of GC markers *Blimp1*, *Stella* and *Fragilis* were enriched for H3K4me3 indicating the transcriptionally active chromatin state, as seen for *Oct3/4* (Supplementary Fig. S2A). In contrast the promoter regions of *Dazl* and *MVH* were decorated with both H3K4me3 and H3K27me3, highlighting the bivalent chromatin state, which is a hall mark of lineage specification genes, such as *Hoxa11* and *Pax5* (Supplementary Fig. S2B). To further validate these observations, gene specific histone modification profiles (active: H3K4me3, H3K9ac; and repressive: H3K9me3, H3K27me3) were analyzed by ChIP at the promoter regions of GC markers *Fragilis* and *Blimp1*, and PrM markers *Dazl* and *MVH*, and compared to the promoter regions of *Oct3/4* (transcriptionally active chromatin) and *Hoxa11* and *Pax5* (bivalent chromatin) in ES cells (Fig. 4A). qPCR quantification of ChIP DNA showed that the promoter regions of GC markers *Fragilis* and *Blimp1* were enriched for the activating modifications H3K4me3 and H3K9ac, but depleted for the repressive modifications H3K9me3 and H3K27me3, indicating a transcriptionally active chromatin similar to key pluripotency *Oct3/4* gene promoter (Fig. 4A). In contrast, the promoters of PrM genes *Dazl* and *MVH* were enriched for both active (H3K4me3 and H3K9ac) and repressive (H3K27me3) modifications, representing the bivalent chromatin domain similar to lineage specific genes (*Hoxa11* and *Pax5*) (Fig. 4A). Moreover, we also performed gene specific histone modification profiling in established iPS cells [20] and found similar results like ES cells (Fig. 4B).

### GC markers emerge during early reprogramming of MEFs into iPSCs

**Figure 5:**
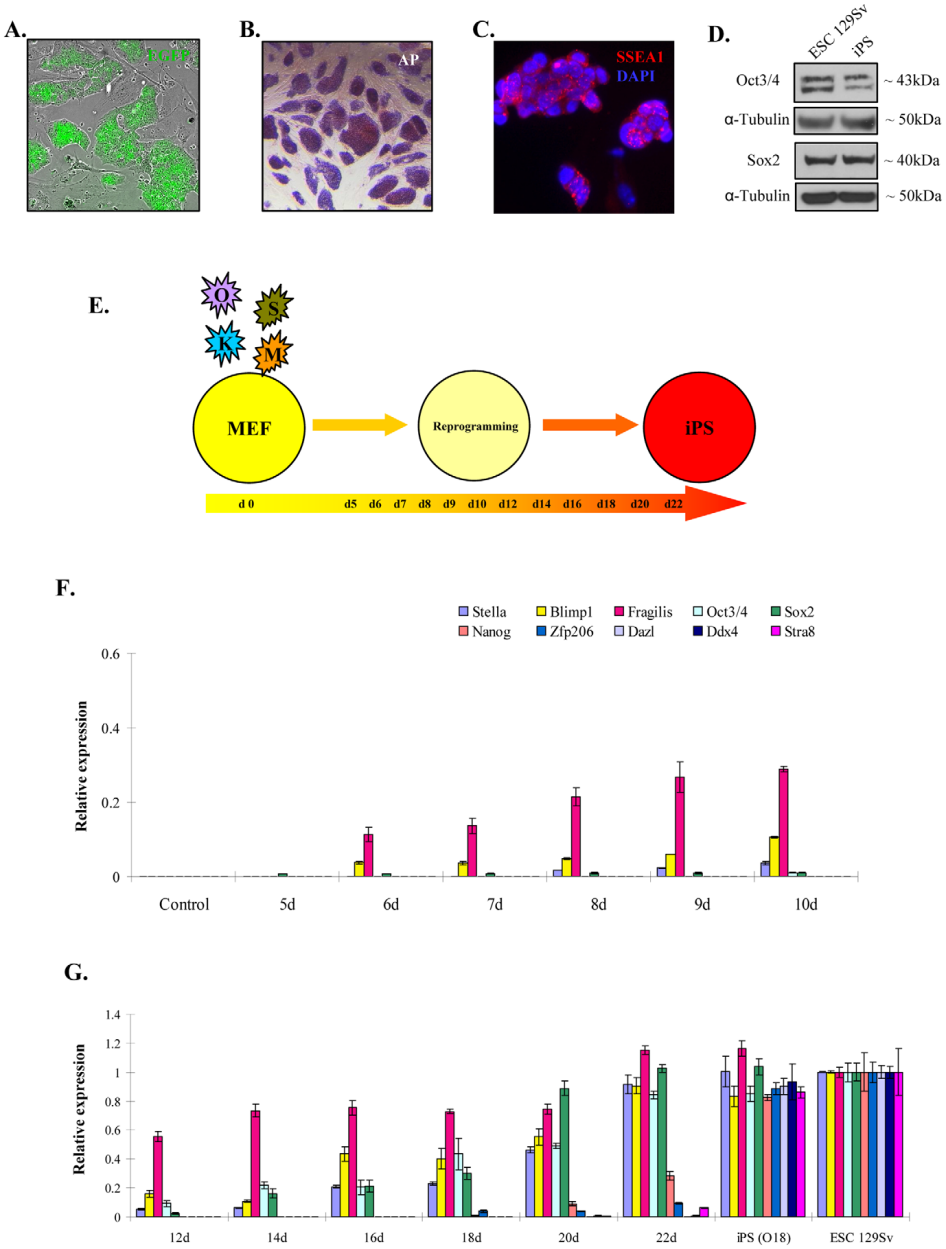
Expression pattern of GC/PrM and pluripotency genes during the course of iPS generation. Fully reprogrammed and established iPS cells express *Nanog* promoter-driven EGFP (**A**), positive for AP staining (**B**), and express SSEA1 (**C**). (**D**) Expression of key pluripotency markers Oct3/4 and Sox2 in iPS cells. Protein extract from ESCs was used as a positive control. (**E**) Schematic diagram depicting the generation of iPS cells using the four Yamanaka factors and the time course of sample collection (starting from the day of retrovirus transduction, day0 (d0) till day22 (d22)) for gene expression analysis. (**F**) Real time qPCR analysis of pluripotency (*Oct3/4*, *Sox2*, *Nanog* and *Zfp206*), germ cell (*Stella*, *Blimp1* and *Fragilis*) and pre-meiotic genes (*Dazl*, *MVH* and *Stra8*) during the time course of iPS cell generation from day5 (5d) to day 10 (10d) after virus infection. *Nanog*-EGFP MEFs were used as a control. **(G)** Real time qPCR analysis for the above mentioned genes during the time course of iPS cell generation from day12 (12d) to day 22 (22d) after virus infection. The qPCR data of two or more biological replicates (including three technical replicates each) were calculated and represented as a mean ±SD.

To further understand the role of GC/PrM markers during the establishment and maintenance of pluripotency, we used ectopic expression of the four Yamanaka factors (Oct4, c-Myc, Klf4, and Sox2) for reprogramming of somatic cells to induced pluripotency. Firstly, we reprogrammed MEFs isolated from Nanog-EGFP mice and established four iPS lines (xu1, 2, 5, and 6), which are morphologically similar to ES cells, activate the *Nanog* promoter-driven EGFP expression, positive for AP staining, SSEA1 immunostaining and express endogenous Oct3/4 and Sox2 (Fig. 5A–D). The iPSC lines were further characterized by histone modification, and DNA methylation profiling of key pluripotent marker genes (data not shown). Finally, all the iPSC lines (xu1, 2, 5 and 6) were injected subcutaneously into immunodeficient mice. Two recipients were used per cell line. In all mice, tumors were observed and the mice had to be sacrificed between day 18 and day 33 after injection. The tumors were identified as teratomas by histological examination as exemplified for iPS cell lines xu2 and 6 (Supplementary Fig. S3). Then, we setup a time course experiment and analyzed the expression levels of key pluripotency genes and GC/PrM genes during the course of reprogramming as outlined in figure 5E. Expression analysis using real time qPCR revealed significant expression levels of key germ cell markers (*Blimp1* and *Fragilis*) at day 6 and a gradual increase to the levels seen in ES cells by day 22 (Fig. 5F, G). Transcripts of *Stella*, another germ cell marker, were significantly detectable at day 10 of reprogramming and reached levels similar to those in ES cells by day 22. (Fig. 5F, G). In contrast, significant endogenous expression levels of the key pluripotency markers *Oct3/4* and *Sox2* occurred only on day 12 of reprogramming and showed expression levels typical for ES cells by day 22 (Fig. 5G). Further pluripotency markers like *Zfp206* and *Nanog* appeared only on day 18 and 20, respectively and increased to levels observed in ES cells only in fully reprogrammed and established iPS cells (Fig. 5G). Surprisingly, we could not detect significant expression of pre-meiotic markers such as *Stra8*, *Dazl* and *MVH* before day 22 of reprogramming. The expression of *Stra8* appeared not until day 22 and the other two markers were only present in established iPS cells (Fig. 5G).

## Discussion

Though traditionally pluripotent ES cells are regarded as *in-vitro* counterpart of the inner cell mass (ICM), their origin is not yet clearly defined. Recently, it has been hypothesized that ESCs may have a germ-cell origin based on common molecular properties with other pluripotent cells of germ-cell origin [9]. Previously, ES cells were shown to express several GC/PrM markers [15], [16], [17]. In agreement with these results, that demonstrated the expression of several GC/PrM markers at the transcript level, our Western blot analysis detected the expression of GC/PrM markers in all analyzed pluripotent cell types available including iPS cells and female ES cells of mouse origin. Consistent with an earlier report [27], expression of GC/PrM genes was not detectable in bone marrow-derived multipotent stem cells, thus indicating the unique expression of GC/PrM genes only in pluripotent cells. Our immunocytochemistry expression analysis of GC/PrM markers in preimplantation embryos revealed the expression of Stella, Dazl and MVH in all analyzed preimplantation embryo stages (2-, 4-, 8-cell stage). Further, down-regulation of PrM genes in ESCs did not influence the expression levels of pluripotency network genes, but rather increase expression of GC genes. Conversely, down-regulation of pluripotency marker Oct3/4 showed no significant effect on GC/PrM marker genes, thus highlighting the maintenance of parallel but independent networks.

The genome-wide expression profiling of ES cells revealed the expression of a large number of genes at low levels due to the open chromatin state of ES cells leading to leaky expression [28], [29], [30], [31]. To elucidate leaky versus essential expression of GC/PrM markers in ES cells, we analyzed the global ChIP-Seq data of ES cells and found an active chromatin state at PGC/germ cell markers and a bivalent chromatin structure at pre-meiotic markers [26]. In support of global ChIP-seq data, our gene-specific chromatin state of GC/PrM markers in ES cells confirmed the active chromatin state with enrichment for the activating histone modifications H3K4me3 and H3K9ac at the promoter regions of PGC markers *Blimp1* and *Fragilis*, which demonstrates the fundamental expression of these genes. In contrast, the promoter regions of *Dazl* and *MVH* were marked with bivalent chromatin state, i.e. enrichment for the two activating (H3K4me3 and H3K9ac) and the repressive (H3K27me3) histone modifications, which is a hallmark of key developmental regulation/lineage specific genes [32], [33]. The observed active chromatin state at GC marker genes might indicate the possible early germ cell specification epigenetic marks in pluripotent cells. Conversely, the bivalent chromatin state at PrM marker genes might represent the poised germ cell lineage specification or the heterogeneous expression of these genes in pluripotent cells.

Recent advances in direct reprogramming of somatic cells to induced pluripotency opened new avenues not only for tailor-made patient-specific cells for future regenerative medicine in addition to advancing our knowledge of the basic biology of establishment and maintenance of pluripotency [34]. Of particular interest is the role of GC/PrM markers during iPS cell generation using the four Yamanaka's factors. We analyzed the activation of GC/PrM markers along with the endogenous activation of core pluripotency markers during somatic reprogramming and found the activation of the PGC specification markers *Blimp1*, *Stella* and *Fragilis* to occur much earlier (between day 6 and day 9 of reprogramming) than activation of the endogenous pluripotency markers *Oct3/4* and *Sox2* (by day12 of reprogramming). In contrast, the expression of the PrM markers *Dazl*, *MVH* and *Stra8* was only detectable by day 22 and in established iPS cell lines, respectively. Recent studies of the molecular mechanisms underlying somatic reprogramming revealed that somatic cells undergo mesenchymal to epithelial transition (MET) during early reprogramming to acquire pluripotency through BMP signaling and vital expression of E-cadherin [35], [36]. Interestingly, during embryonic development, PGC precursors rely on inductive BMP signals followed by MET activation and Fragilis, Blimp1, Stella and E-cadherin expression [37], [38]. Loss of BMP signals, Blimp1 and E-cadherin expression results in the depletion or a reduced number of PGCs [38], [39], [40], [41]. Taken together, we assume that even somatic cells acquire a “temporary” PGC/GC fate and finally establish pluripotency during reprogramming.

**Figure 6:**
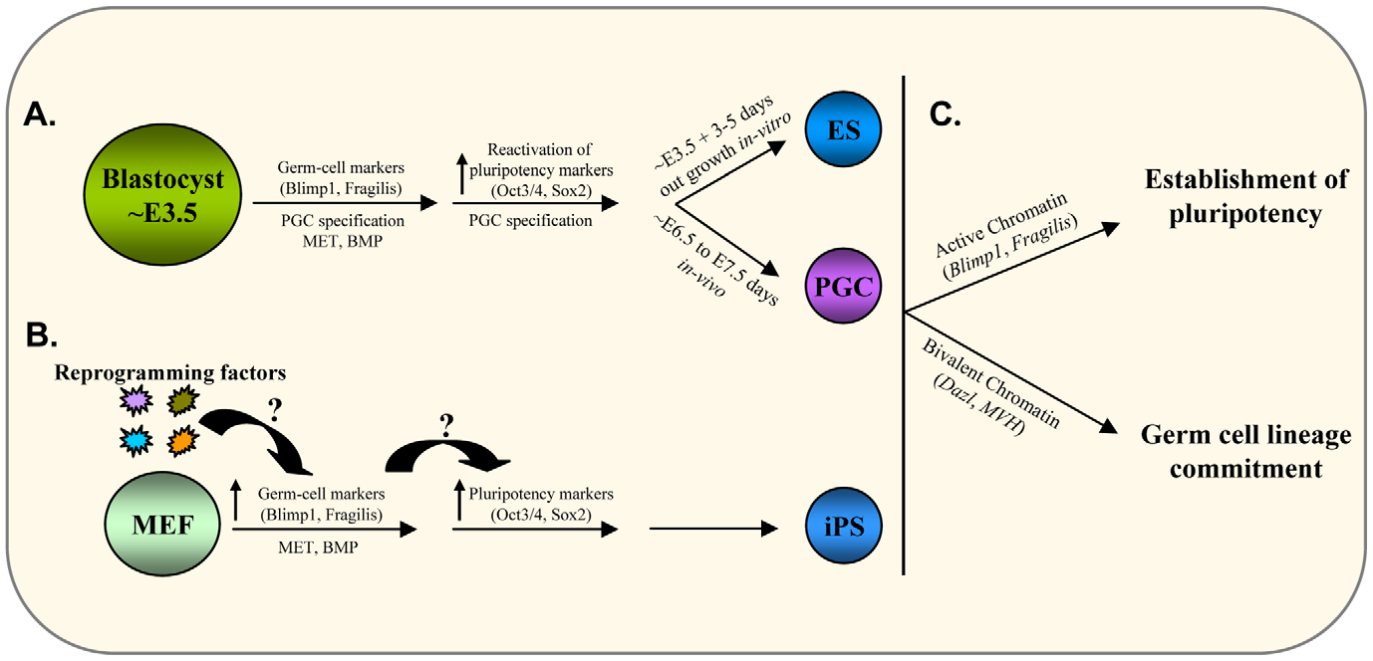
Hypothetical model for the germ cell origin of pluripotent ESCs. (**A**) The inner cell mass cells of the blastocyst are positive for Oct3/4 and Sox2 expression. During further development, primordial germ cell (PGC) specification in mouse implantation embryos (blastocyst (∼E3.5) stage onwards) is marked by the expression of germ cell markers Blimp1 and Fragilis followed by reactivation of Oct3/4 and Sox2 and is completed by ∼E6.5-E7.5 *in vivo*. This period of *in vivo* PGC specification parallels with the *in vitro* ESCs generation from pre-implantation blastocysts (∼E3.5) together with 3–5 days of ESCs outgrowth. (**B**) On the other hand, reprogramming of somatic cells to iPS cells using the four Yamanaka factors (Oct3/4, Sox2, Klf4 and c-Myc) leads to the early appearance of germ cell markers followed by the activation of endogenous Oct3/4 and Sox2 and subsequent establishment of pluripotent state. This pattern of gene activation is equivalent to that of PGC specification and ESC establishment as discussed above. (**C**) The active chromatin state of germ cell markers in ESCs might indicate the developmental origin of ESCs from PGCs; the presence of bivalent chromatin at the promoter regions of pre-meiotic genes indicate the poised state for germ line commitment.

Based on our study and earlier reports [9], we propose a working model for the germ-cell origin of ESCs and the possible acquisition of PGC/GC fate by somatic cells during iPSCs generation (Fig. 6). According to our model, the ICM of blastocyst stage embryos (∼E3.5) expresses key pluripotency markers Oct3/4, Sox2, and c-Myc. Following embryonic development, PGC specification *in-vivo* is marked by the expression of key PGC genes, where Blimp1 is activated by BMP signaling [10], facilitates the activation of Stella and E-cadherin, initiates the repression of the somatic program, and reactivates the pluripotency network before PGCs acquire migratory properties [38] (Fig. 6A). Considering the GC fate and lineage commitment of PGCs, key germ-cell markers may have active chromatin, whereas PrM genes may show bivalent chromatin (Fig. 6C). Similarly, ESC generation also starts with isolation of ∼E3.5 blastocysts followed by culture to obtain outgrowth from the ICM. It is more likely that during the *in-vitro* ICM outgrowth, ICM cells proceed with the pre-programmed developmental program of PGC specification via BMP signals, initiate MET, begin expressing Fragilis, Blimp1, and Stella, re-activate pluripotency genes, and acquire the unique self-renewal property (Fig. 6A). The observed active chromatin state of *Blimp1*, *Stella,* and *Fragilis* thus might indicate the unique expression or PGC/GC origin of ESC and the bivalent chromatin state of *Dazl* and *MVH* confers the germ-cell lineage commitment, as has been observed for other lineages (Fig. 6C). Similarly, during somatic reprogramming, addition of Oct3/4, Sox2, c-Myc, and Klf4 to somatic cells might mimic the *in-vivo* ∼E3.5 blastocyst ICM cells and follows the induction of BMP signaling and hence the activation of Fragilis, Blimp1, Stella, and E-cadherin, and MET (Fig. 6B). Further, activation of the endogenous pluripotency network from the host cell genome finally establishes pluripotent cell characteristics (Fig. 6B). Finally, the chromatin state of GC/PrM markers may also reflect their transition through germ-cell fate (Fig. 6C).

In summary, we show the expression of GC/PrM markers in all analyzed pluripotent cell types and show parallel but independent maintenance of GC/PrM networks from pluripotent networks. Through our data, we propose a hypothetical model for possible germ-cell origin of ESCs and suggest the plausible transition of somatic cells through germ-cell fate to achieve pluripotency. Further genetic and epigenetic studies aimed at PGC specification during ICM outgrowth may resolve and increase our knowledge of pluripotency.

## Supporting Information

Figure S1 **Effect of the downregulation of Oct3/4 in ES cells.** (**A**) Real time qPCR demonstrating the down-regulation of *Oct3/4* and the expression profile of other pluripotency markers (*Klf4*, *Nanog*, *Zfp206* and *Lin28*). (**B**) Expression profile of germ cell (*Stella* and *Fragilis*) and pre-meiotic (*Dazl*, *MVH* and *Stra8*) markers in *Oct3/4* down-regulated ESCs. The dotted lines indicate the normalized expression levels of analyzed genes in control siRNA treated cells. The qPCR data of two biological replicates (including three technical replicates each) were calculated and represented as a mean ±SD. Expression levels, which are statistically significant, are indicated with asterisks (***p<0.001).
(TIF)

Figure S2 **Epigenetic signature of pluripotency and GC/PrM genes in ES cells.** (**A**) Chip-seq data from the database showing that the promoter regions (red box) of pluripotency marker gene *Oct3/4* and germ cell markers *Blimp1* (*Prdm1*), *Stella* (*Dppa3*) and *Fragilis* (*Ifitm3*) representing open chromatin with abundance of active histone modification H3K4me3 (green peaks) and are depleted of repressive marks like H3K27me3 and H3K9me3 (highlighted with red and brown peaks respectively). In contrast, the promoter regions of pre-meiotic genes *MVH* (*Ddx4*), *Dazl*, *Hoxa11* and *Pax5* were marked with both active and repressive histone modification marks, signifying their bivalent chromatin structure (**B**).
(TIF)

Figure S3 **Histopathological analysis identifies iPS cell-derived tumors as teratomas.** Tumors grown in RAG2^−/−^cγc^−/−^ mice after injection of iPS cell lines #xu2 and #xu6 were HE stained. The tumors are teratomas showing ectodermal mesodermal and endodermal differentiations (* skin epithelium, # cartilage, → muscle, ▸ gut epithelium). The scale bar represents 100 µm.
(TIF)

Table S1 **Primers used in RT-PCR.**
(DOC)

Table S2 **Quantitative real-time PCR primers for siRNA down regulation study.**
(DOC)

Table S3 **Quantitative real-time PCR primers used to test endogenous gene expression.**
(DOC)

Table S4 **List of antibodies used in Western blotting.**
(DOC)

Table S5 **Quantitative real-time PCR primers used in Chip assay.**
(DOC)
