# Supplementary figures and images for: Stage-Specific Germ-Cell Marker Genes Are Expressed in All Mouse Pluripotent Cell Types and Emerge Early during Induced Pluripotency
Source: PLoS One. 2011 Jul 25;6(7):e22413. doi: 10.1371/journal.pone.0022413 (PMC3143132; doi:10.1371/journal.pone.0022413)

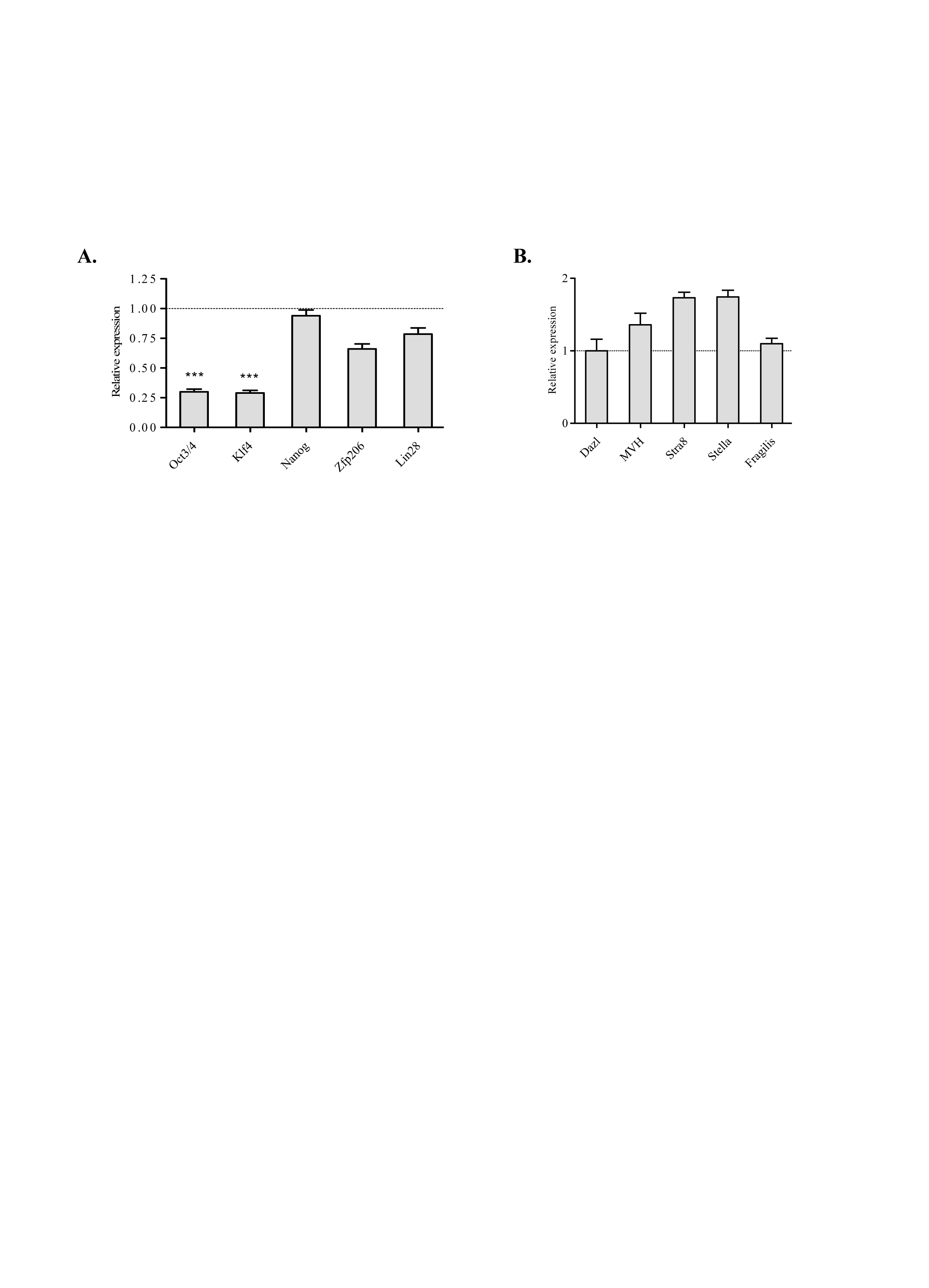

Supplement: Figure S1 — Effect of the downregulation of Oct3/4 in ES cells. (A) Real time qPCR demonstrating the down-regulation of Oct3/4 and the expression profile of other pluripotency markers (Klf4, Nanog, Zfp206 and Lin28). (B) Expression profile of germ cell (Stella and Fragilis) and pre-meiotic (Dazl, MVH and Stra8) markers in Oct3/4 down-regulated ESCs. The dotted lines indicate the normalized expression levels of analyzed genes in control siRNA treated cells. The qPCR data of two biological replicates (including three technical replicates each) were calculated and represented as a mean ±SD. Expression levels, which are statistically significant, are indicated with asterisks (***p<0.001). (TIF) [file pone.0022413.s001.tif]

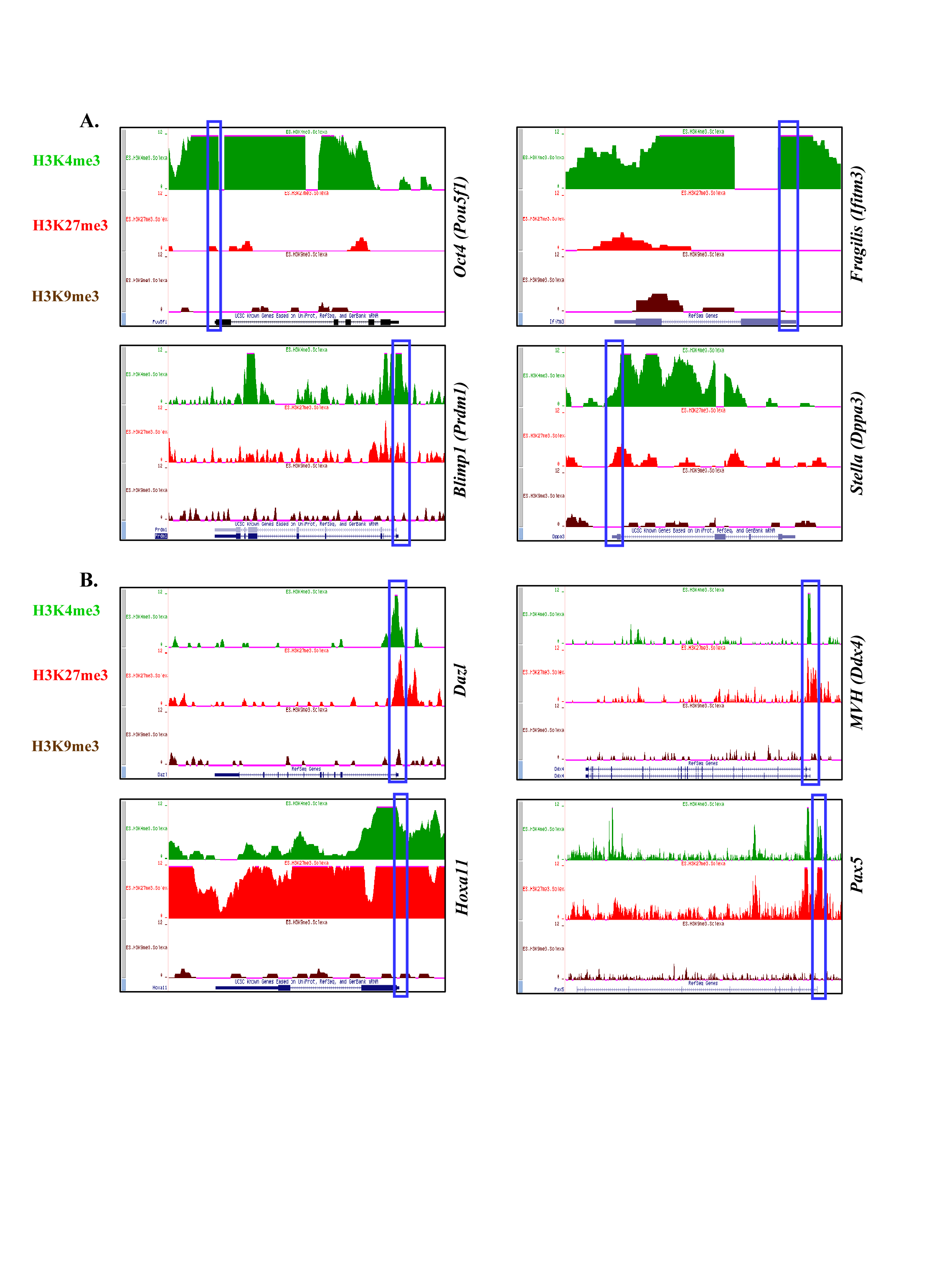

Supplement: Figure S2 — Epigenetic signature of pluripotency and GC/PrM genes in ES cells. (A) Chip-seq data from the database showing that the promoter regions (red box) of pluripotency marker gene Oct3/4 and germ cell markers Blimp1 (Prdm1), Stella (Dppa3) and Fragilis (Ifitm3) representing open chromatin with abundance of active histone modification H3K4me3 (green peaks) and are depleted of repressive marks like H3K27me3 and H3K9me3 (highlighted with red and brown peaks respectively). In contrast, the promoter regions of pre-meiotic genes MVH (Ddx4), Dazl, Hoxa11 and Pax5 were marked with both active and repressive histone modification marks, signifying their bivalent chromatin structure (B). (TIF) [file pone.0022413.s002.tif]

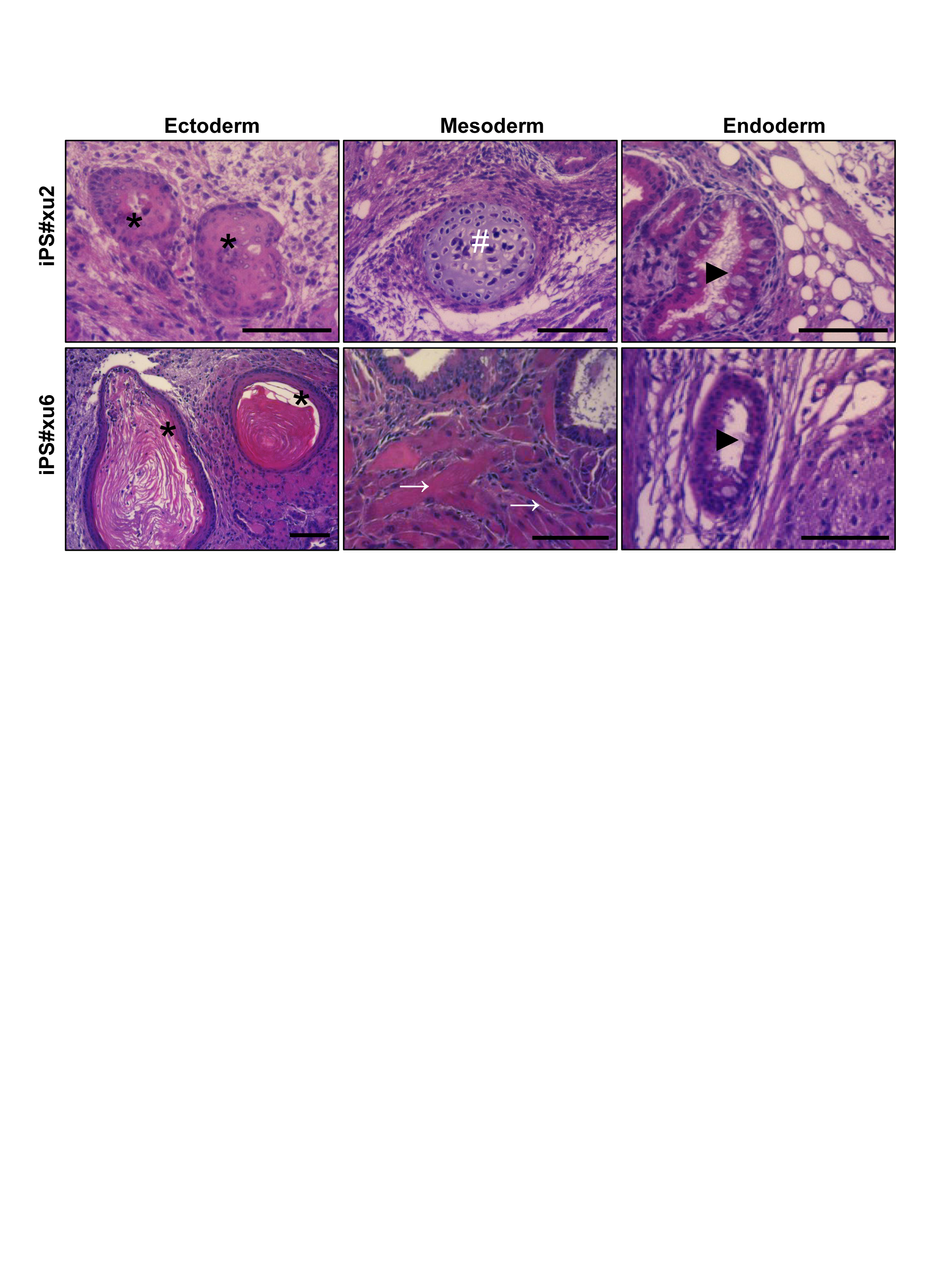

Supplement: Figure S3 — Histopathological analysis identifies iPS cell-derived tumors as teratomas. Tumors grown in RAG2−/−cγc−/− mice after injection of iPS cell lines #xu2 and #xu6 were HE stained. The tumors are teratomas showing ectodermal mesodermal and endodermal differentiations (* skin epithelium, # cartilage, → muscle, ▸ gut epithelium). The scale bar represents 100 µm. (TIF) [file pone.0022413.s003.tif]
