# Supplementary material for: Stage-Specific Germ-Cell Marker Genes Are Expressed in All Mouse Pluripotent Cell Types and Emerge Early during Induced Pluripotency
Source: PLoS One. 2011 Jul 25;6(7):e22413. doi: 10.1371/journal.pone.0022413 (PMC3143132; doi:10.1371/journal.pone.0022413)
Supplement: Table S1 — Primers used in RT-PCR. (DOC) [file pone.0022413.s004.doc]

**Supplementary tables**

**Table S1. Primers used in RT-PCR**

| **Gene** | **Forward primer sequence** | **Reverse primer sequence** |
| --- | --- | --- |
| Creb3/4 | 5’-CAGAAACATCTCCTGGTAGGG-3’ | 5’-GGGCAGAGTAATCCCTTCTTG-3’ |
| Cylc1 | 5’-GATCAGGACCTTCAGAATTAG-3’ | 5’-CCAGATTTACTACACATGAGC-3’ |
| Gpx4 | 5’-CGCCAAAGTCCTAGGAAACGC -3’ | 5’-CAGGCAGACCATGTGCCCGTC -3’ |
| Hprt | 5’-CGTCGTGATTAGCGATGATG-3’ | 5’-TATGTCCCCCGTTGACTGAT-3’ |
| Pgk2 | 5’-TCTCATGAGTCACCTCGGTCG-3’ | 5’-AACTGTGAGCCCGATGTGCAG-3’ |
| Piwil2 | 5’-GCACAGTCCACGTGGTGGAAA-3’ | 5’-TCCATAGTCAGGACCGGAGGG-3’ |
| Prm1 | 5’-CCCACAAAATTCCACCTGCTC-3’ | 5’-CAGGTGGCATTGTTCCTTAGC-3’ |
| Rnf17 | 5’-GACACACAGTCTAACAGAGG-3’ | 5’-AGGACAGCAGCATCTACCTT-3’ |
| Rnh2 | 5’-CATAAGTGGCAACGAAGAGC-3’ | 5’-GTTACAGGCTGCTACCATCA-3’ |
| Stra8 | 5’-TCACAGCCTCAAAGTGGCAGG-3’ | 5’-GCAACAGAGTGGAGGAGGAGT-3’ |
| Sycp3 | 5’-GTTGCAGCAGTGGGAACTGG-3’ | 5’-CTAAAGGCATGCCTCTTAGC-3’ |
| Theg | 5’-GCTCAGTAACCCAATCCCGGAGGT-3’ | 5’-GGATGGACCAGATGGGCGTTGTCC-3’ |
| Tp2 | 5’-CGGCCTCAAAGTCACACCAGT-3’ | 5’-AGTCCGTTTCCGCCTCCTGAC-3’ |
