# Supplementary material for: Stage-Specific Germ-Cell Marker Genes Are Expressed in All Mouse Pluripotent Cell Types and Emerge Early during Induced Pluripotency
Source: PLoS One. 2011 Jul 25;6(7):e22413. doi: 10.1371/journal.pone.0022413 (PMC3143132; doi:10.1371/journal.pone.0022413)
Supplement: Table S2 — Quantitative real-time PCR primers for siRNA down regulation study. (DOC) [file pone.0022413.s005.doc]

**Table S2. Quantitative real-time PCR primers for siRNA down regulation study**

| **Gene** | **Forward primer sequence** | **Reverse primer sequence** |
| --- | --- | --- |
| Dazl | 5’-CTCCACCTTCGAGGTTTTACC-3’ | 5’-CTGATTTCGGTTTCATCCATC-3’ |
| MVH | GAGAAGTGGGTTTCCTTCTGG | 5’-GAAAACCCTCTGCTTCGAGTC-3’ |
| Fragilis | 5’-AACATGCCCAGAGAGGTGTC-3’ | 5’-CTTAGCAGTGGAGGCGTAGG-3’ |
| Hnf4 | 5’-CCACATGTACTCCTGCAGGTTTAG-3’ | 5’-CGCTCATTTTGGACAGCTTC-3’ |
| Hprt | 5’- AGCCCCAAAATGGTTAAGGTTGC-3’ | 5’- TTGCAGATTCAACTTGCGCTCAT-3’ |
| Klf4 | 5’-TCAGGTACCCCTCTCTCTTCTTC-3’ | 5’-CGCTTCATGTGAGAGAGTTCCT-3’ |
| Lin 28 | 5’-GCATCTGTAAGTGGTTCAACGTG-3’ | 5’-TCACTCCCAATACAGAACACACC-3’ |
| Nanog | 5’-TTACAAGGGTCTGCTACTGAGTG-3’ | 5’-CAGGACTTGAGAGCTTTTGTTTG-3’ |
| Nestin | 5’-CGCTGGAACAGAGATTGGAGG-3’ | 5’-CCTCCAGCAGAGTCCTGTATG-3’ |
| Oct3/4 | 5’-CGGAAGAGAAAGCGAACTAGC-3’ | 5’-GCCTCATACTCTTCTCGTTGG-3’ |
| Sdha | 5’-GCTTGCGAGCTGCATTTGG-3’ | 5’-CATCTCCAGTTGTCCTCTTCCA-3’ |
| Stella | 5’-CCCTGAAACTCCTCAGAAGAAAG-3’ | 5’-TTTTCACTCTTGTTCTCCACAGG-3’ |
| Stra8 | 5’-GTGTTCCACAAGTGTCGAAGGT-3’ | 5’-GGAGTAAACTGCCTTCCTTAGGT-3’ |
| Vimentin | 5’-TGCAGTCATTCAGACAGGATGT-3’ | 5’-ATCTCTTCATCGTGCAGTTTCTTC-3’ |
| Zfp206 | 5’-GAGAGGAGGTGGTACAGCTATTG-3’ | 5’-AGGTGGAGGTAACTCATTCAGTG-3’ |
| Blimp1 | 5’-CACACAGGAGAGAAGCCACA-3’ | 5’-TTGTGACACTGGGCACACTT-3’ |
