# Supplementary material for: Stage-Specific Germ-Cell Marker Genes Are Expressed in All Mouse Pluripotent Cell Types and Emerge Early during Induced Pluripotency
Source: PLoS One. 2011 Jul 25;6(7):e22413. doi: 10.1371/journal.pone.0022413 (PMC3143132; doi:10.1371/journal.pone.0022413)
Supplement: Table S3 — Quantitative real-time PCR primers used to test endogenous gene expression. (DOC) [file pone.0022413.s006.doc]

**Table S3. Quantitative real-time PCR primers used to test** endogenous gene expression

| **Gene** | **Forward primer sequence** | **Reverse primer sequence** |
| --- | --- | --- |
| Nanog | 5’-CCAGGTTCCTTCCTTCTTCC-3’ | 5’-GGTGAGATGGCTCAGTGGAT-3’ |
| Oct3/4 | 5’-TAGGTGAGCCGTCTTTCCAC-3’ | 5’-GCTTAGCCAGGTTCGAGGAT-3’ |
| Sox2 | 5’-CTGGACTGCGAACTGGAGAA-3’ | 5’-CTAGTCGGCATCACGGTTTT-3’ |
