# Supplementary material for: Stage-Specific Germ-Cell Marker Genes Are Expressed in All Mouse Pluripotent Cell Types and Emerge Early during Induced Pluripotency
Source: PLoS One. 2011 Jul 25;6(7):e22413. doi: 10.1371/journal.pone.0022413 (PMC3143132; doi:10.1371/journal.pone.0022413)
Supplement: Table S4 — List of antibodies used in Western blotting. (DOC) [file pone.0022413.s007.doc]

| **Antibody** | **Source** |
| --- | --- |
| Dazl | ab34139 (Abcam) |
| MVH | ab13840 (Abcam) |
| Dmc1 | ab11054 (Abcam) |
| Fragilis | ab15592 (Abcam) |
| Oct3/4 | ab19857 (Abcam) |
| Piwil2 | ab36764 (Abcam) |
| Sox2 | ab15830 (Abcam) |
| SSEA1 | ab16285 (Abcam) |
| Stella | ab19878 (Abcam) |
| α-Tubulin | Sig T5168 (Sigma) |
| Histone H3 (tri methyl K9) | ab8898 (Abcam) |
| Histone H3 (tri methyl K27) | ab6002 (Abcam) |
| Histone H3 (tri methyl K4) | ab8580 (Abcam) |
| Histone H3 (acetyl K9) | ab4441 (Abcam) |

**Table S4. List of antibodies used in Western blotting**
