# Supplementary material for: Stage-Specific Germ-Cell Marker Genes Are Expressed in All Mouse Pluripotent Cell Types and Emerge Early during Induced Pluripotency
Source: PLoS One. 2011 Jul 25;6(7):e22413. doi: 10.1371/journal.pone.0022413 (PMC3143132; doi:10.1371/journal.pone.0022413)
Supplement: Table S5 — Quantitative real-time PCR primers used in Chip assay. (DOC) [file pone.0022413.s008.doc]

| **Gene** | **Forward primer sequence** | **Reverse primer sequence** |
| --- | --- | --- |
| Blimp1 | 5’-GAGCGAGCGACTGACTACTCTT-3’ | 5’-ATCCATTTGCAAACAGAGGAAG-3’ |
| Dazl | 5’-CTACGTGAGGTGGCTGCCTA-3’ | 5’-CTATTGGCTGTAGCACGTCAC-3’ |
| MVH | 5’-GCGGCTTAAACGGCTGCAC-3’ | 5’-GCCTCAACAAAGGTGGAGAA-3’ |
| Fragilis | 5’-GGTTTCTCAGAAGTTTCCTTTTCC-3’ | 5’-TCACTCTTAAACACTGAGGTTTGG-3’ |
| Hoxa11 | 5’-AGAAGTGCCTCTGGCTCTGA-3’ | 5’-GATTTGCACGGTGACTTGATT-3’ |
| Oct3/4 | 5’-TGGGCTGAAATACTGGGTTC-3’ | 5’-TTGAATGTTCGTGTGCCAAT-3’ |
| Pax5 | 5’-ATGGGAGTTTGTTTTCCTGTGT-3’ | 5’-ATGGGAGTTTGTTTTCCTGTGT-3’ |

**Table S5. Quantitative real-time PCR primers used in Chip assay**
